# BYD Ameliorates Oxidative Stress-Induced Myocardial Apoptosis in Heart Failure Post-Acute Myocardial Infarction via the P38 MAPK-CRYAB Signaling Pathway

**DOI:** 10.3389/fphys.2018.00505

**Published:** 2018-05-08

**Authors:** Yi Zhang, Chun Li, Hui Meng, Dongqing Guo, Qian Zhang, Wenji Lu, Qixin Wang, Yong Wang, Pengfei Tu

**Affiliations:** ^1^Modern Research Center for Traditional Chinese Medicine, School of Chinese Materia Medica, Beijing University of Chinese Medicine, Beijing, China; ^2^The School of Life Sciences, Beijing University of Chinese Medicine, Beijing, China; ^3^College of Chinese Medicine, Beijing University of Chinese Medicine, Beijing, China

**Keywords:** BYD, oxidative stress, apoptosis, P38 MAPK-CRYAB, HF post-AMI

## Abstract

**Aim:** Heart failure (HF) post-acute myocardial infarction (AMI) contributes to increasing mortality and morbidity worldwide. Baoyuan decoction (BYD) is a well-known traditional Chinese medicine formula that exhibits myocardial protection clinically. The aim of this study was to identify the effects of BYD on oxidative stress-induced apoptosis in HF post-AMI and characterize the underlying mechanism.

**Methods and Results:** In our study, we constructed left anterior descending (LAD)-induced AMI rat models and a macrophage-conditioned media (CM)-induced H9C2 injury model. *In vivo*, BYD could protect cardiac functions, decrease inflammatory cell infiltration and inhibit oxidative stress-induced apoptosis. *In vitro*, BYD inhibited cellular apoptosis and regulated the expressions of key apoptotic molecules, including reducing the expression of B cell lymphoma-2 (Bcl-2) associated X protein (Bax) and cleaved caspase-3 and -9. Interestingly, the P38 mitogen-activated protein kinase (MAPK)-αB-crystallin (CRYAB) signaling pathway was activated by BYD treatment, and the P38 MAPK inhibitor SB203580 could reverse the protective effects of BYD.

**Conclusion:** This study identified that BYD protected against oxidative stress-induced myocardial apoptosis via the P38 MAPK-CRYAB pathway. CRYAB may become a novel therapeutic target for AMI.

## Introduction

Acute myocardial infarction (AMI) is a severe cardiovascular disease that can lead to heart failure (HF) and malignant arrhythmia, which are responsible for increasing mortality and morbidity worldwide (Plakht et al., 2015; Sacks et al., 2015). Currently, the focus has been on elucidating the underlying mechanisms and exploring new therapeutic targets for HF post-AMI.

Accumulating evidence has demonstrated that oxidative stress-induced apoptosis is an attractive target for HF post-AMI (Shahzad et al., 2017). Following acute ischaemia, the dynamic balance between the oxidation system and anti-oxidation system is broken, and multiple reactive oxygen species (ROS) gather (Riba et al., 2017). As a major intracellular source of ROS, mitochondria are directly damaged by increased ROS, which affect the mitochondrial permeability (Circu and Aw, 2010). Consequently, B cell lymphoma-2 (Bcl-2) associated X protein (Bax), caspase 3, caspase 9 related to the mitochondrial apoptotic pathway are activated, and Bcl-2, which has an anti-apoptotic effect against the mitochondrial apoptotic pathway, is inhibited. As a result, apoptosis is induced dramatically with the consequence of damaged heart function (Ryter et al., 2007).

CRYAB, which encodes αB-crystallin, a member of the small heat shock protein family, was first considered the conserved structure of the cytoskeleton (Bennardini et al., 1992). Until recently, some studies have suggested that it exhibits a remarkable anti-oxidative stress (OS)-induced apoptotic effect in HF post-AMI, and CRYAB phosphorylation Ser-59 is selectively responsible for the cytoprotective actions in cardiac myocytes (Kamradt et al., 2001; Morrison et al., 2003; Mao et al., 2004; Taylor and Benjamin, 2005). Increasing oxidative stress will lead to the up-regulation of p-CRYAB, which can protect cardiomyocytes from OS-induced apoptosis. Consistently, CRYAB silencing resulted in increased apoptosis after exposure to OS (Chis et al., 2012). Some studies have indicated that CRYAB plays an anti-apoptotic role by down-regulating Bcl-2 expression in H_2_O_2_-injured H9C2 cells (Xu et al., 2013). Further studies demonstrated that CRYAB, phosphorylated by the P38 mitogen-activated protein kinase (MAPK) cascade, played an anti-apoptotic role against stress-induced apoptosis in cardiac myocytes (Hoover et al., 2000).

Baoyuan decoction (BYD) is a well-known traditional Chinese medicine formula composed of *Astragalus, ginseng, liquorice* and *cinnamon* (Wan et al., 2017). BYD has been approved for myocardial protection clinically (Zhang et al., 2016), however, the potential pharmacological mechanism remains unclear. In this study, we investigated the cardiac protective effects of BYD on an oxidative stress-induced apoptosis in HF post-AMI rat model and conditioned media (CM)-induced H9C2 cell model and clarified the underlying mechanism of BYD in the P38 MAPK-CRYAB signaling pathway.

## Materials and Methods

### BYD Preparation

The four components of BYD were collected from Anguo TCM market (Hebei, China) and were authenticated by Professor Pengfei Tu. The extraction method was performed as reported previously (Ma et al., 2016). The fingerprint of BYD was analyzed by high-performance liquid chromatography (HPLC), and the typical chromatogram was shown in a previous study (Shu et al., 2016).

### HF Post-AMI Animals

All animal experiments conformed to the Guide for the Institutional Animal Care and Use Committee (IACUC) and were approved by the Animal Care Committee of Beijing University of Chinese Medicine.

Eighty male Sprague-Dawley (SD) rats weighing 220–250 g in the SPF grade were purchased from Beijing Vital River Laboratory Animal Technology Co., Ltd. (Beijing, China). The left anterior descending coronary artery (LAD) of the rats was ligated to construct the HF post-AMI models as previously described (Wang et al., 2012, 2015). Briefly, left thoracotomy was performed between the third and fourth intercostal space, and the heart was exposed. The LAD was ligated just 1–2 mm proximal to the main diagonal branch with a sterile suture (Shuangjian, China). The chest was closed, and the rats were placed on a thermal blanket. In the sham group, the suture was passed around the artery without ligation. Twenty-four hours after surgery, the rats were randomly divided into six groups: sham group, model group, Ginaton Tablets group (as the positive drug with a dosage of 100 mg/kg; Dr. Willmar Schwabe, Germany), BYD high-dose group (BYD-h, with a dosage of 2.57 g/kg), BYD middle-dose group (BYD-m, with a dosage of 1.28 g/kg) and BYD low-dose group (BYD-l, with a dosage of 0.64 g/kg). Ginaton Tablets were applied as the positive-control drug because it could inhibit OS and suppress apoptosis by regulating P38 MAPK (Li et al., 2017; Tang et al., 2017). The rats in the Ginaton Tablets group and BYD groups were administered via gavage with the described doses for 7 days. The sham and model groups received the same volume of water through oral administration.

### Echocardiography

After anaesthetization with 1% pentobarbital sodium (50 mg/kg, ip), the left ventricular function was assessed by 2-dimensional M-mode and B-mode echocardiography (Vevo TM 2100; Visual Sonics, Canada). The indicators of the left ventricular internal diameter at end-diastole (LVIDd), left ventricular internal diameter at end-systole (LVIDs), left ventricular end of diastole volume (LVEDV), left ventricular end of systole volume (LVESV), ejection fraction (EF), fractional shortening (FS), and others were collected. FS% was calculated using the following equation: FS% = [(LVIDd-LVIDs)/LVIDd] × 100%. EF% was calculated using the following equation: EF% = [(LVEDV-LVESV)/LVEDV] × 100%.

### Haemodynamic Measurement

After echocardiography, haemodynamic measurements were performed to evaluate the performance of left ventricular (LV). The levels of the mean blood pressure (MBP), maximal rate of increase of the left ventricular pressure (*+dp/dt max*) and maximal rate of decrease of the left ventricular pressure (*-dp/dt max*) were recorded using the PowerLab ML880 system (AD Instrument, Australia).

### Histological Examination and Immunohistochemistry Detection

Myocardial tissues were fixed in 4% paraformaldehyde for 72 h and then were embedded in paraffin and sectioned into 5-μm slices. The paraffin sections were subjected to haematoxylin-eosin (HE) staining and were analyzed under an optical microscope at 400 × magnification.

Paraffin sections were deparaffinized and immersed in distilled water. There after, the sections were blocked with 3% hydrogen peroxide at room temperature for endogenous peroxidase ablation for 15 min, followed by blockade in normal goat serum at room temperature for 20 min. After discarding the goat serum, the sections were incubated with rabbit anti-CRYAB phosphor-S59 (ab5577; Abcam, United States) at 4°C overnight, followed by incubation with goat anti-rabbit IgG (ab16284; Abcam, United States) for 1 h at room temperature. After colouration with 3, 3-diaminobenzidin (DAB) and staining with haematoxylin, the sections underwent dehydration, clearing and mounting with neutral gums. Images were visualized and collected under an optical microscope at 400 × magnification.

### Detection of LDH, CK-MB, T-SOD and MDA

Serum samples were collected from the abdominal aorta after centrifugation at 3,000 rpm for 10 min. The levels of creatine kinase-MB (CK-MB) in the serum was determined using a Hitachi 17080 Automatic Biochemical Analyzer (Hitachi Co., Ltd., Japan), and lactate dehydrogenase (LDH) was tested using commercial diagnostic kits (Beijing North Biotechnology Research Institute, China). The levels of total superoxide dismutase (T-SOD) and malondialdehyde (MDA) were detected according to the manufacturer’s instructions of the T-SOD assay kit and MDA assay kit, respectively (Nanjing Jiancheng Bioengineering Institute, China).

### Terminal Deoxynucleotidyl Transferase Mediated dUTP Nick End Labeling (TUNEL) Staining

The apoptosis rate was determined by TUNEL according to the manufacturer’s instructions (Roche Applied Science; South San Francisco, CA, United States). Micrographs were randomly selected and analyzed.

### Western Blot Analysis

Heart tissues or cells were lysed in pre-cold RIPA buffer (Beijing Pulilai Gene Technology Co., Ltd., China) with a 1% protein phosphatase inhibitor (Beijing Pulilai Gene Technology Co., Ltd., China). The protein concentration in each sample was measured by the BCA kit (Beijing Pulilai Gene Technology Co., Ltd., China). The samples were loaded on 10% SDS-PAGE gels and were transferred onto PVDF membranes. The membranes were first incubated overnight with primary antibody at 4°C and secondary antibody 1.5 h at room temperature and then were treated with ECL (ECL Plus western blotting detection reagent, GE Healthcare, United States) for 1 min at room temperature. The following antibodies were used: anti-CRYAB phosphor-S59 (ab5577; Abcam, United States), anti-CRYAB (ab13497; Abcam, United States), anti-phospho MAPK-activated protein kinase 2 (MAPKAPK2; 3041; Cell Signaling Technology, Germany), anti-MAPKAPK2 (3042; Cell Signaling Technology, Germany), anti-phospho MKK 3/MKK6 (9236; Cell Signaling Technology, Germany), anti-MKK6 (8550; Cell Signaling Technology, Germany), anti-phospho P38 MAPK (4511; Cell Signaling Technology, Germany), anti-P38 MAPK (8690; Cell Signaling Technology, Germany), anti-Caspase 3 (9665; Cell Signaling Technology, Germany), anti-Caspase 9 (9580; Cell Signaling Technology, Germany), anti-Bax (ab32503; Abcam, United States), anti-Bcl-2 (ab7973; Abcam, United States), anti-GAPDH (ab8245; Abcam, United States), anti-rabbit IgG H&L (HRP; ab16284; Abcam, United States), anti-mouse IgG H&L (HRP; ab97250; Abcam, United States).

### Cell Culture

RAW 264.7 macrophages and H9C2 cells were purchased from China Infrastructure of Cell Line Resources (Institute of Basic Medical Sciences, Chinese Academy of Medical Sciences). They were incubated with Dulbecco’s modified Eagle’s medium (DMEM; Corning, United States) supplemented with 10% fetal bovine serum (FBS, Corning, United States), penicillin (100 U/mL; Corning, United States) and streptomycin (100 μg/mL; Corning, United States) at 37°C in a humidified atmosphere of 5% CO_2_.

### Cell Viability

Cell Counting Kit-8, a commercially available cell viability assay, was employed to evaluate the cytotoxic effect. Ten percent CCK-8 (Dojindo, Kumamoto, Japan) dissolved in DMEM was added to each well for 2 h at 37°C, and absorbance was determined at 450 nm using a microplate reader (Perkin-Elmer, Waltham, MA, United States). The percentage of cell viability was calculated by the following formula: cell viability (%) = (mean absorbance in test wells)/(mean absorbance in control wells) × 100%. All experiments were performed in triplicate (Manual, 2010).

### LPS-Induced RAW 264.7 Cell Injury

RAW 264.7 cells were divided into different groups: control, model, BYD groups (with 400, 600, 800, and 1000 μg/mL, respectively) and Ginaton Tablets group (40 μg/mL, as positive control drug). After the RAW 264.7 cells were cultured to 95% confluency in plates, different concentrations of BYD or Ginaton Tablets in culture media were added into the cells. Next, lipopolysaccharide (LPS; 1 μg/mL; Sigma Chemical Co., United States) was added and stimulated for 24 h. The control group was given the same operation without drugs or LPS. Cell supernatants were collected and stored at -20°C for further analysis. Next, 1 × 10^5^ cells/well of RAW 264.7 cells were seeded into 96-well plates for the cell viability test. For western blot analysis and determination of ROS, 2 × 10^6^ cells/well of RAW 264.7 cells were seeded in 6-well plates.

### CM-Induced H9C2 Cell Injury

To evaluate the effects of BYD on H9C2 cells, the model of macrophage-conditioned media (CM) stimulation was performed as previously described (Li et al., 2016). Flow diagram of this model was showed in **Figure 5A**. CM was collected from the supernatant liquid of RAW 264.7 cells pretreated with 1 μg/mL LPS for 24 h. After culture to 95% confluence in plates, H9C2 cells were pretreated with BYD (400, 600, 800, and 1000 μg/mL) for 6 h, and then cells were incubated with CM for 24 h. Cell supernatants were collected and stored at -20°C for further analysis. To investigate the activation mechanism of CRYAB, SB203580 (10 μM) was added to H9C2 for 1 h, followed by stimulation with CM for 24 h.

### Measurement of NO and T-SOD

Cell supernatants were collected from LPS-stimulated RAW 264.7 cells (with or without BYD pretreatment) to detect NO released from cultured cells. NO production was determined using the NO assay kit (Biyuntian Biotechnology Co., Ltd., China) based on the Griess method using a microplate reader (Perkin-Elmer, Waltham, MA, United States).

Cell supernatants were collected from CM-induced H9C2 cells (with or without BYD pretreatment) to detect T-SOD expressions. The expressions of T-SOD were determined using the T-SOD assay kit (Nanjing Jiancheng Bioengineering Institute, China), following the manufacturer’s instructions.

### Hoechst Staining and Reactive Oxygen Species Measurement

H9C2 cells were fixed with 4% paraformaldehyde for 15 min and were stained with Hoechst 33258 (Biyuntian Biotechnology Co., Ltd., China) for 30 min in the dark. Next, H9C2 cells were observed under an inverted fluorescence microscope (Leica Microsystems GmbH).

The ROS assay was conducted according to the manufacturer’s instructions of the ROS Assay Kit bought from Biyuntian Biotechnology Co., Ltd. Next, 10 μM DCFH diluted in DMEM was added to each well, followed by incubation for 30 min at 37°C. The cells were then observed under an inverted fluorescence microscope (Leica Microsystems GmbH).

### Statistical Analysis

The results were presented as the means ± SEM. Statistical analysis of the data was carried out using one-way analysis of variance (ANOVA) and Dunnett’ s test. *P < 0.05* was considered statistically significant.

## Results

### BYD Rescues the Cardiac Functions of HF Post-AMI Rats

**FIGURE 1:**
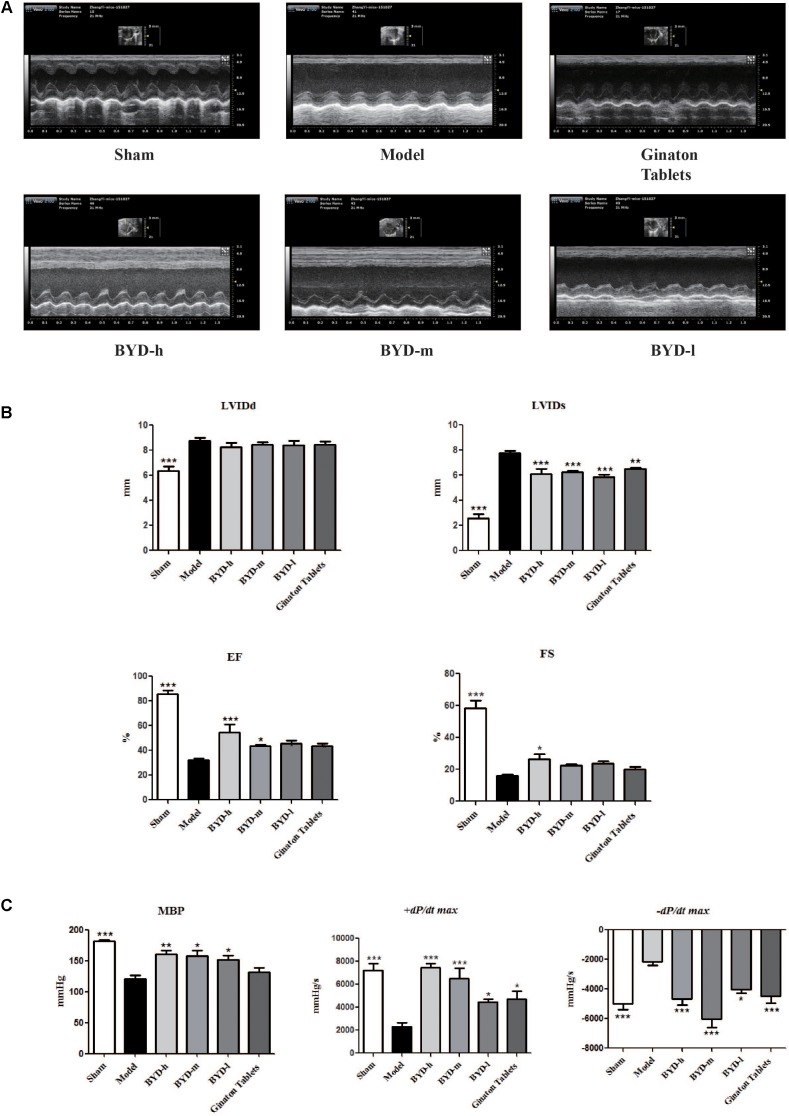
BYD improves cardiac function. **(A)** Representative images of echocardiography exhibiting the changes in cardiac function in each group. **(B)** Echocardiography results showed that BYD improved EF and FS and decreased LVIDs. **(C)** Haemodynamic results showed that BYD up-regulated the levels of MBP and *+dp/dt max* and down-regulated the level of *–dp/dt max*, suggesting that BYD could improve cardiac contraction function. All the data were presented as the means ± SEM from independent experiments performed in triplicate. ^∗^*P* < 0.05, ^∗∗^*P* < 0.01, ^∗∗∗^*P* < 0.001 vs. model group. *N* = 10 per group.

Echocardiography results showed that HF post-AMI models were successfully constructed. The levels of EF and FS were significantly decreased in the model group compared with those in the sham group (*P* < 0.001). Moreover, LVIDd and LVIDs were increased in the model group (*P* < 0.001), indicating that both heart dysfunction and structural change occurred. MBP and *+dp/dt max* were decreased and *-dp/dt max* was increased in the model group (*P* < 0.001), suggesting impairment of diastolic and systolic LV functions. After treatment with BYD, the values of EF and FS were up-regulated (*P* < 0.001, *P* < 0.05, respectively), and LVIDs was also improved significantly (*P* < 0.001); however, there was no statistically significant difference in LVIDd before and after BYD treatment (**Figures 1A,B**). Compared with the sham group, BYD treatment in different doses increased the level of MBP (*P* < 0.01) and *+dp/dt max* (*P* < 0.001) and decreased the levels of *-dp/dt max* (*P* < 0.001) (**Figures 1C**). Ginaton Tables showed similar efficacy with BYD.

### Cardioprotective Effects of BYD in HF Post-AMI Rats

**FIGURE 2:**
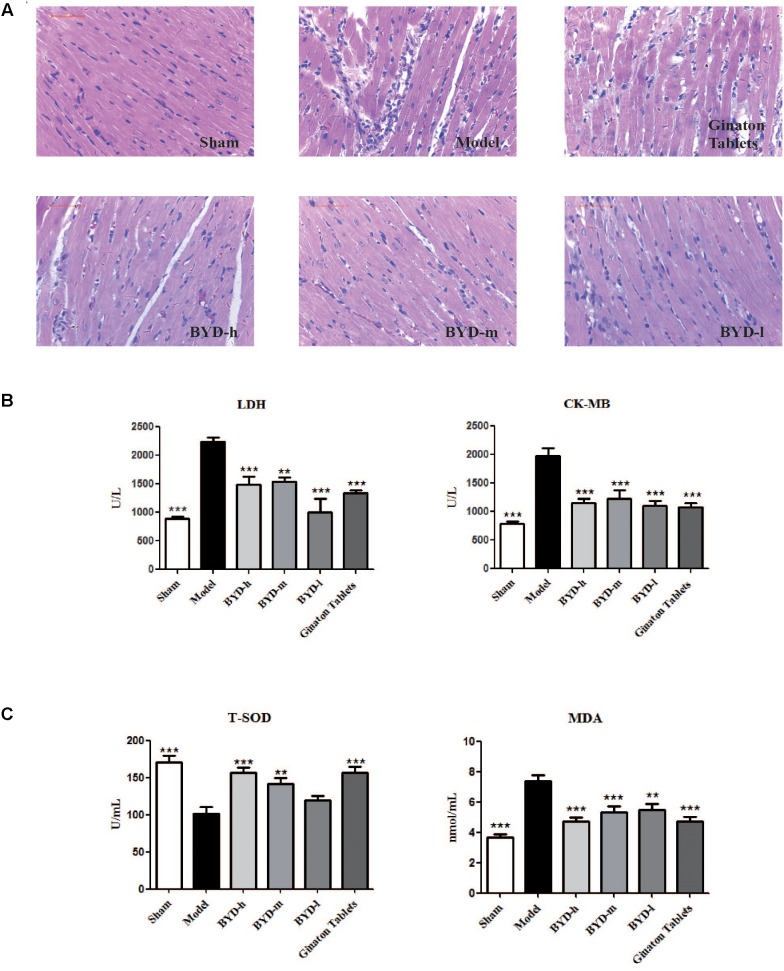
BYD inhibits inflammatory cell infiltration and protects against cardiac injury. **(A)** HE showed that BYD preserved cardiomyocyte architecture and inhibited inflammatory cell infiltration. **(B)** The concentrations of LDH and CK-MB in serum in the model group were increased compared with those in the sham group and could be up-regulated by BYD. **(C)** BYD treatment up-regulated the level of T-SOD and down-regulated the level of MDA in serum compared with model group. All the data were presented as the means ± SEM from independent experiments performed in triplicate. ^∗∗^*P* < 0.01, ^∗∗∗^*P* < 0.001 vs. the model group. *N* = 6 per group.

Inflammatory cell infiltration and cellular morphology were detected by HE staining (**Figure 2A**). Large necrotic areas with inflammatory cell infiltration could be observed in the model group. The inflammatory response plays an important role in HF post-AMI rats. The LV myocardium was arranged in an orderly pattern in the sham group, whereas the surviving cardiomyocytes formed an irregular pattern in the model group. Treatment with different doses of BYD could reduce inflammatory cell infiltration and partially restore the cardiomyocyte damage.

### Effects of BYD on the Serum LDH, CK-MB, T-SOD and MDA Levels

LDH and CK-MB are located in the cytoplasm of cardiomyocytes under normal conditions. The release of LDH and CK-MB into the blood is considered diagnostic indicators of HF post-AMI (Amani et al., 2013). The results showed that the levels of serum LDH and CK-MB in the model group were significantly up-regulated compared with those in the sham group (*P* < 0.001, *P* < 0.001). After treatment with BYD, the LDH and CK-MB levels were reduced compared with those in the model group (*P* < 0.001, *P* < 0.001, respectively) (**Figure 2B**). T-SOD is a remarkable anti-oxidant enzyme, while MDA is one of the final products of OS, and both are commonly used as markers for OS (Ribeiro-Samora et al., 2017). BYD treatment increased the activity of T-SOD but attenuated the MDA level compared with that in the model group (*P* < 0.001, *P* < 0.001, respectively) (**Figure 2C**).

### BYD Attenuates Oxidative Stress in LPS-Induced RAW 264.7 Cells

**FIGURE 3:**
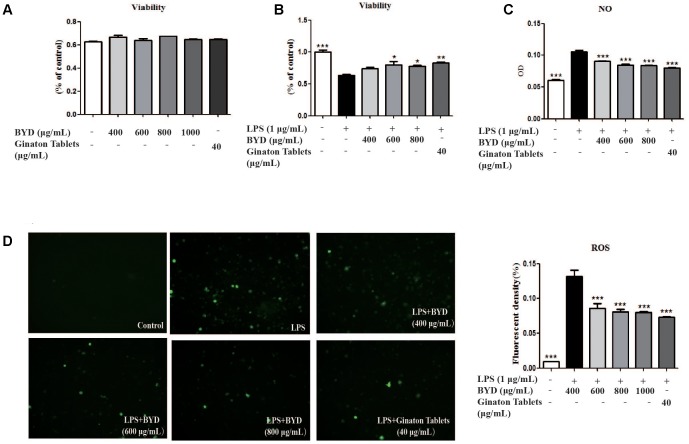
BYD regulates the levels of OS mediators *in vitro*. **(A)** The doses involved in this study showed no cytotoxicity at 24 h. **(B)** BYD showed a protective effect on LPS-induced RAW 264.7 cells. **(C)** BYD significantly decreased the release of NO induced by LPS. **(D)** The results of ROS determination showed that BYD treatment decreased the green fluorescent intensity. All the data were presented as the means ± SEM from independent experiments performed in triplicate. ^∗^*P* < 0.05, ^∗∗^*P* < 0.01, ^∗∗∗^*P* < 0.001 vs. the model group. *N* = 6 per group.

To confirm whether the effects of BYD on HF post-AMI rats were associated with anti-oxidative stress, the LPS-induced injury model in RAW 264.7 macrophage was applied. Treatment of RAW 264.7 with BYD at 400–1000 μg/mL and positive control drug Ginaton Tablets (40 μg/mL) showed no cytotoxicity after 24 h (**Figure 3A**). Interestingly, CCK-8 results demonstrated BYD exhibited significant cell-protective effects against LPS-induced injury (*P* < 0.05) (**Figure 3B**). Moreover, BYD and Ginaton Tablets treatment could decrease the production of ROS and NO (*P* < 0.001) *in vitro* (**Figures 3C,D**).

### BYD Inhibits Apoptosis in HF Post-AMI Rats via the CRYAB Signaling Pathway

**FIGURE 4:**
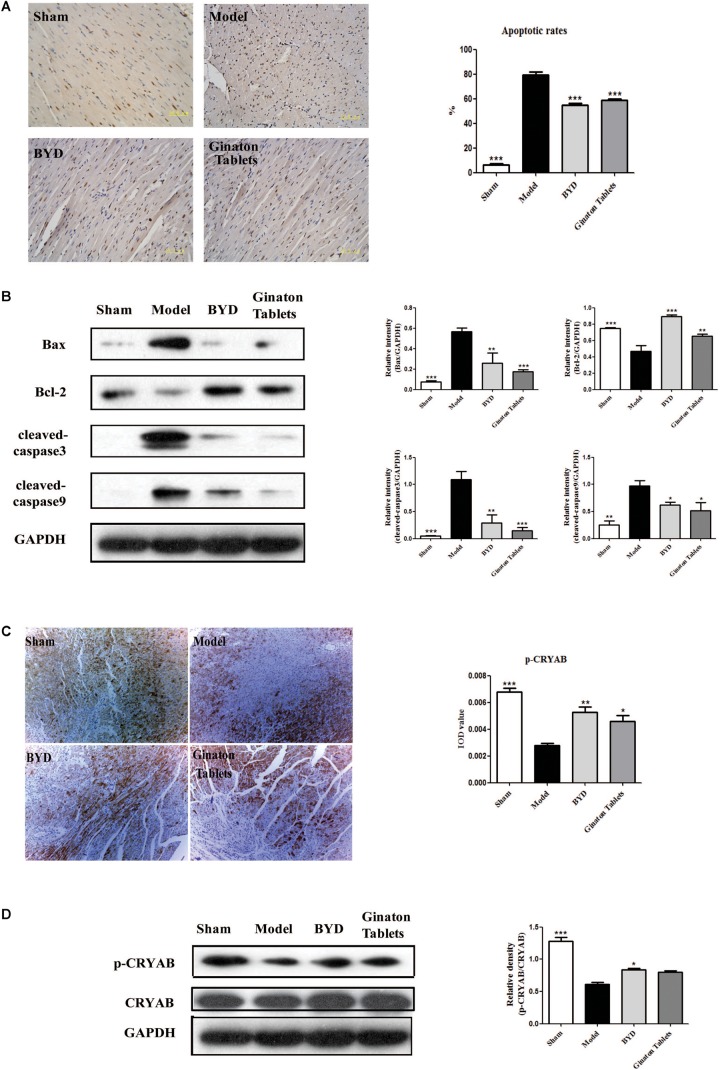
BYD inhibited apoptosis and increased CRYAB in HF post-AMI rats. **(A)** TUNEL results showed that BYD attenuated the apoptosis of cardiomyocytes. **(B)** Western blot analysis showed that BYD decreased the expression of Bax, cleaved-caspase 9 and cleaved-caspase 3 and increased the expressions of Bcl-2 in cardiac tissue compared with that in the model group. **(C)** Immunohistochemistry results showed that BYD up-regulated the expression of p-CRYAB in cardiac tissue. **(D)** Western blot analysis results showed that BYD up-regulated the expression of p-CRYAB in cardiac tissue compared with that in the model group. The same GAPDH band was selected due to reusing the membrane by stripping solution. All the data were presented as the means ± SEM from independent experiments performed in triplicate. ^∗^*P* < 0.05, ^∗∗^*P* < 0.01, ^∗∗∗^*P* < 0.001 vs. the model group. N = 3 per group. The original bands of Western blot were showed in Supplementary Figure 1.

TUNEL staining showed that the apoptotic rate in the infarct border zone was significantly higher in the model group than in the sham group, while BYD treatment could reverse the increased apoptosis rate in the model group (**Figure 4A**). Further study on the upstream proteins of the apoptosis pathway showed that BYD inhibited the expression of Bax (*P* < 0.01), cleaved-caspase 9 (*P* < 0.05) and cleaved-caspase 3 (*P* < 0.01) and increased the expression of Bcl-2 (*P* < 0.001) compared with that in the model group (**Figure 4B**). As mentioned above, CRYAB is believed to be a critical anti-apoptosis protein that regulates cardiomyocyte apoptosis induced by ischemia (Mitra et al., 2014). In our study, both immunohistochemistry and western blotting demonstrated that the expression of p-CRYAB was down-regulated in the model group, and BYD could up-regulate p-CRYAB as shown in **Figures 4C,D** (*P* < 0.05).

### BYD Inhibits Apoptosis in CM-Induced H9C2 Cells

**FIGURE 5:**
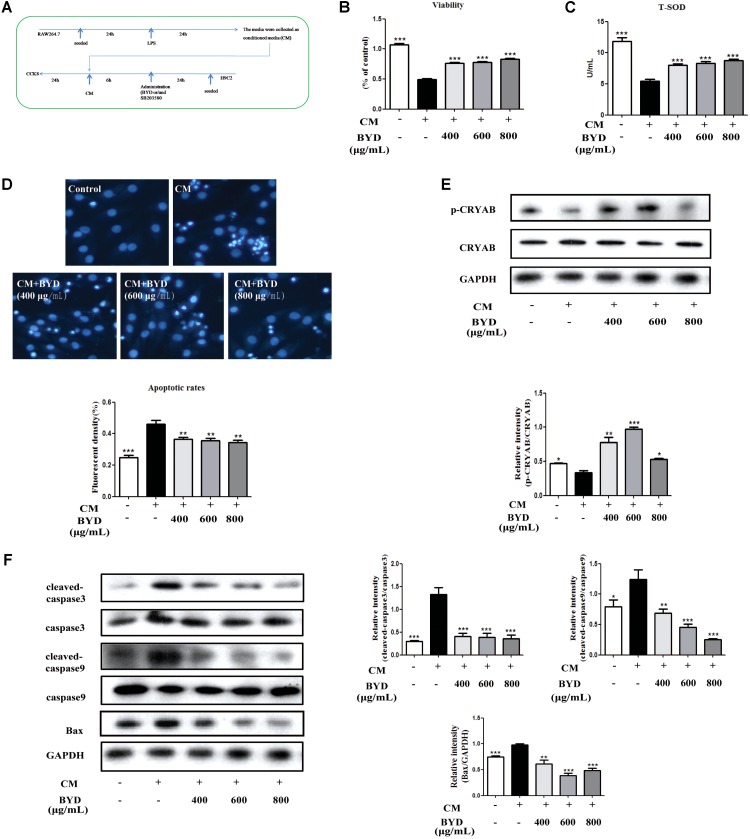
BYD inhibits apoptosis in CM-induced H9C2 cells. **(A)** Flow diagram of CM-induced H9C2 cell injury. **(B)** BYD showed a significant protective effect on CM-induced H9C2 cell injury. **(C)** BYD could increase the release of T-SOD from H9C2 injuried by CM. **(D)** Hoechst 33258 staining results showed that BYD attenuated apoptosis in CM-induced H9C2 cells. **(E)** BYD could up-regulate the expression of p-CRYAB *in vitro*. **(F)** Different doses of BYD treatments down-regulated the increased expression of Bax, cleaved-caspase 9 and cleaved-caspase 3 compared with that in the model group. The same GAPDH band was selected due to reusing the membrane by stripping solution. All the data were presented as the means ± SEM from independent experiments performed in triplicate. ^∗^*P* < 0.05, ^∗∗^*P* < 0.01, ^∗∗∗^*P* < 0.001 vs. the model group. *N* = 3 per group for western blotting and Hoechst 33258 staining. *N* = 6 per group in the CCK-8 and T-SOD test. The original bands of Western blot were showed in Supplementary Figure 2.

To evaluate the effects of BYD on H9C2 cells *in vitro*, CM was used to induce H9C2 apoptosis. CCK-8 results showed that BYD could increase the cell viability in CM-induced H9C2 cells (*P* < 0.001) (**Figure 5B**). The T-SOD test showed that BYD treatment increased the release of T-SOD from H9C2 cells injuried by CM compared with CM-induced H9C2 cells without BYD treatment (**Figure 5C**). The Hoechst 33258 staining assay was performed to determine the effects of BYD on cellular apoptosis. As presented in **Figure 5D**, dying, more condensed or fragmented chromatin was observed in the model group, and BYD treatment could attenuate the apoptosis rate. Western blotting results showed that the expression of p-CRYAB was down-regulated in CM-induced H9C2 cells (*P* < 0.05) but up-regulated in the BYD treatment groups (*P* < 0.05) *in vitro* (**Figure 5E**). Different doses of BYD treatments inhibited the expression of Bax (*P* < 0.01), cleaved-caspase 9 (*P* < 0.01) and cleaved-caspase 3 (*P* < 0.001) in CM-induced H9C2 cells (**Figure 5F**).

### CRYAB Is Activated by the P38 MAPK Cascade Pathway

**FIGURE 6:**
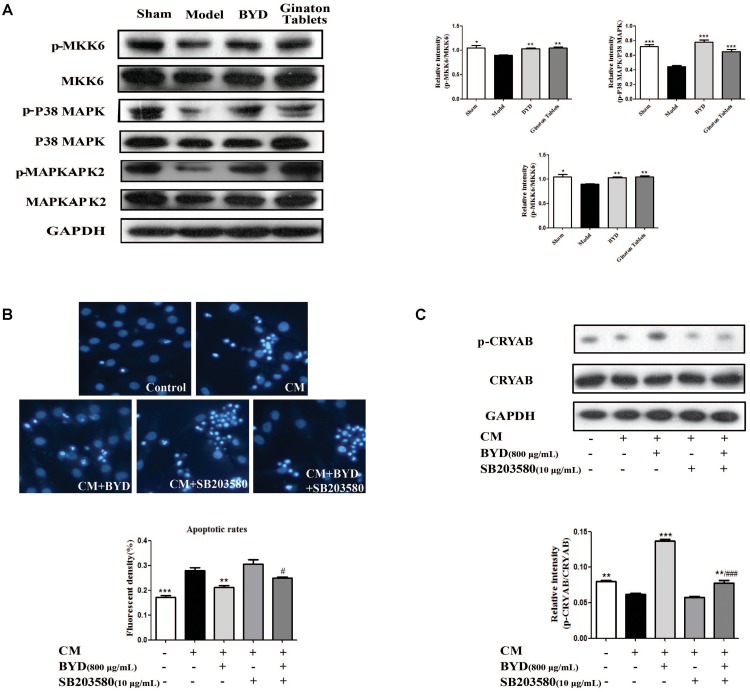
BYD exerts its anti-apoptosis effect through the P38 MAPK-CRYAB pathway. **(A)** BYD activated the P38 MAPK cascade pathway to activate CRYAB *in vivo*. **(B)** The Hoechst 33258 staining assay showed that, after SB203580 treatment, the effect of BYD on anti-apoptosis was inhibited. **(C)** Western blot results showed that, compared with the model group, the expression of p-CRYAB was down regulated in both the SB203580 + model group and BYD + SB203580 group. All the data were presented as the means ± SEM from independent experiments performed in triplicate. ^∗^*P* < 0.05, ^∗∗^*P* < 0.01, ^∗∗∗^*P* < 0.001 vs. model group ^#^*P* < 0.05, ^###^*P* < 0.001 vs. CM+BYD group. *N* = 3 per group. The original bands of Western blot were showed in Supplementary Figure 3.

CRYAB could be activated by the P38 MAPK cascade pathway as previously reported (Mitra et al., 2014). The expression of each protein in the P38 MAPK cascade pathway was detected by western blotting in HF post-AMI rats. The results showed that, compared with the sham group, the expression levels of p-MKK6 (*P* < 0.05), p-P38 MAPK (*P* < 0.001) and p-MAPKAPK2 (*P* > 0.05) were all down-regulated in the model group, consistent with previous studies. Interestingly, BYD treatment could up-regulate the expression of the above proteins compared with that in the model group (*P* < 0.01, *P* < 0.001, *P* < 0.05). Considering the CRYAB results, we assumed that BYD might activate CRYAB via the P38 MAPK cascade pathway (**Figure 6A**).

To validate our hypothesis, the inhibitor of P38 MAPK, SB203580, was used *in vitro*. The results indicated that SB203580 could block the effect of BYD on increasing p-CRYAB, consequently failing to exert an anti-apoptosis effect on the CM-induced H9C2 cells model (**Figures 6B,C**).

## Discussion

In the current study, we mainly focused on whether BYD could attenuate apoptosis induced by oxidative stress via the P38 MAPK-CRYAB pathway. The main findings were as follows: (1) BYD could improve cardiac function, attenuate OS and inhibit apoptosis in HF post-AMI rats; (2) BYD inhibited oxidative stress by decreasing the production of ROS and MDA from cardiac myocytes and LPS-stimulated RAW 264.7 macrophages; (3) BYD protected H9C2 cells against CM-induced apoptosis; (4) *In vivo* and *in vitro* studies suggested that the anti-apoptotic effect of BYD was potentially exerted by regulating the P38 MAPK-CRYAB signaling pathway.

Previous studies have confirmed that OS is a key mechanism to promote the process of HF (Hamilton et al., 2016). ROS, remarkable biomarkers of OS, are highly active molecules that play important roles as “redox messengers” in intracellular signaling and regulation. In the presence of ischemia or hypoxia, the lack of oxygen delivery to myocardium leads to the concentration of ROS increasing rapidly and accumulating (Circu and Aw, 2010). Additionally, T-SOD and MDA are appropriate markers to evaluate OS (Todorova et al., 2005). In our study, the serum level of MDA in the model group was up-regulated, while the serum level of T-SOD was down-regulated, compared with that in the model group. BYD treatment increased the serum activity of T-SOD but attenuated the MDA level compared with that in the model group. *In vitro*, our results also proved that OS production released into media and BYD treatment could inhibit the levels of ROS and increase the expressions of SOD. To some extent, BYD shows a favorable effect on inhibiting MDA and increasing T-SOD to anti-OS both *in vivo* and *in vitro*.

Oxidative stress may be critical for the activation of apoptosis in HF (Bashar and Akhter, 2014). The imbalance between pro-apoptotic proteins (such as Bax, cleaved caspase-3 and -9 and anti-apoptotic proteins (Bcl-2) has long been established as a key determinant in the mitochondrial apoptotic pathway in myocardial apoptosis. Caspases are a family of cysteine-aspartic proteases that play an essential role in pro-apoptosis, while Bcl-2 protein can suppress several apoptotic death programs (Mitupatum et al., 2016). In our study, TUNEL analysis showed that, in the model group, the apoptosis rate was significantly higher than that in the sham group, suggesting that apoptosis was induced. After BYD treatment, the apoptosis rates were significantly reduced and the expression levels of pro-apoptosis proteins, including Bax, caspase 9, and caspase 3 were down-regulated compared with those in the model group, while the expression levels of anti-apoptosis proteins such as Bcl-2 and CRYAB were up-regulated. The results of *in vitro* experiments also further proved that BYD could attenuate apoptosis induced by OS and prevent HF. Additionally, BYD treatment rescued the cardiac function of HF post-AMI rats, including EF, FS, LVIDs, MBP, *+dp/dt max* and *-dp/dt max*.

**FIGURE 7:**
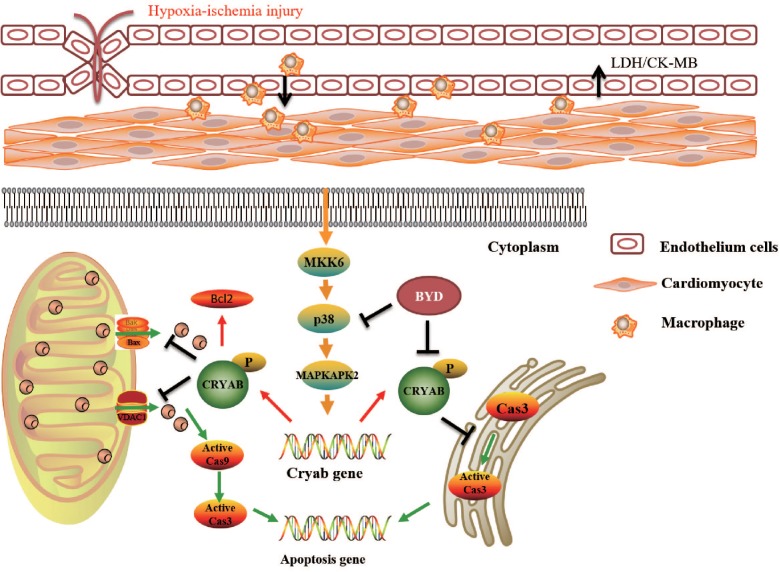
Potential mechanism underlying how BYD exerts a cardioprotective effect.

The mechanism of BYD in inhibiting oxidative stress-induced apoptosis was further investigated. CRYAB, the most abundantly expressed stress protein in the heart, has been demonstrated to play a vital role by interaction with ER stress and the mitochondrial apoptotic pathway during cardiac hypertrophy and myocardial infarction (Mitra et al., 2013). Our study demonstrated that BYD treatment could up-regulate the expression of CRYAB both *in vitro* and *in vivo* by western blotting and immunohistochemistry. P38 MAPK signaling was implicated in the progression of chronic HF. The inhibition of the p38 MAPK pathway protected cardiac function against myocardial infarction in the rat (See et al., 2004). P38 MAPK, which is activated via the MKK6 pathway, stimulates MAPKAPK2, which, in turn, phosphorylates CRYAB. We found that BYD up-regulated the expression levels of p-MKK6, p-P38 MAPK, and p-MAPKAPK2. *In vitro*, SB203580 was used to inhibit the expression of P38 MAPK. Compared with the model group, the cellular apoptosis rate was increased with SB203580 treatment. BYD treatment up-regulated the expression levels of p-MKK6, p-P38 MAPK, p-MAPKAPK2, and CRYAB compared with those in the model group and inhibitor group. Therefore, BYD might partly activate the P38 MAPK cascade to phosphorylate CRYAB and finally exert an anti-apoptosis effect in the HF post-AMI model (**Figure 7**).

## Conclusion

In conclusion, our study evaluated the anti-apoptotic effects of BYD both *in vivo* and *in vitro*, and the mechanism is mediated by regulation of the P38 MAPK-CRYAB signaling pathway. Our findings also provide new insight to further understand the pharmacological mechanism of BYD and alternative strategies for HF post-AMI treatment.

## Author Contributions

YZ and CL performed the research, analyzed the data, and wrote the manuscript. HM and QZ contributed to animal experiments. WL and QW contributed to echocardiography and cell culture. DG, YW, and PT designed and funded the research, revised the manuscript, and approved the submission of this manuscript. All authors have read and agreed with the manuscript.

## Conflict of Interest Statement

The authors declare that the research was conducted in the absence of any commercial or financial relationships that could be construed as a potential conflict of interest.
