# Supplementary material for: BYD Ameliorates Oxidative Stress-Induced Myocardial Apoptosis in Heart Failure Post-Acute Myocardial Infarction via the P38 MAPK-CRYAB Signaling Pathway
Source: Front Physiol. 2018 May 8;9:505. doi: 10.3389/fphys.2018.00505 (PMC5951999; doi:10.3389/fphys.2018.00505)

Supplementary Figures

BYD ameliorates oxidative stress-induced myocardial apoptosis in heart failure post-acute myocardial infarction via the P38 MAPK-CRYAB signalling pathway

Yi Zhang[1#], Chun Li[1#], Hui Meng[1], Dongqing Guo[2], Qian Zhang[2], Wenji Lu[3], Qixin Wang[1], Yong Wang[2*], Pengfei Tu[1*]

Supplementary Figure 1: Full gel images for Figure 4

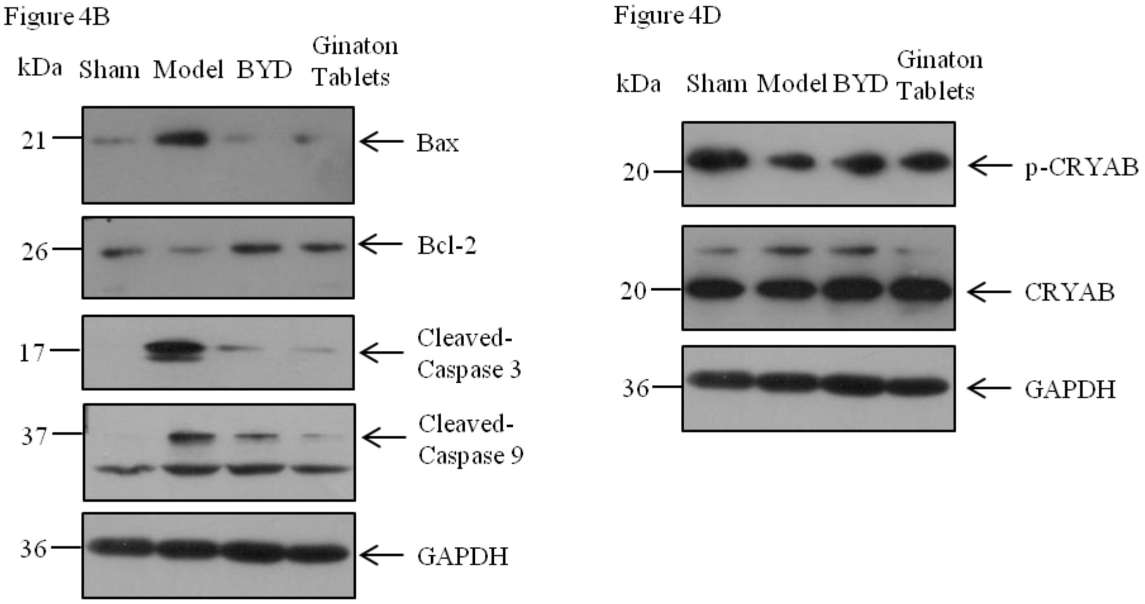

Supplementary Figure 2: Full gel images for Figure 5

Figure 5E

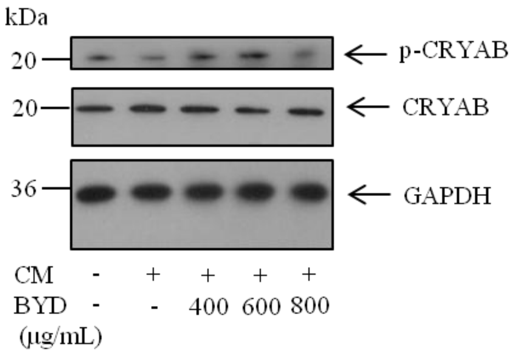

Figure 5F

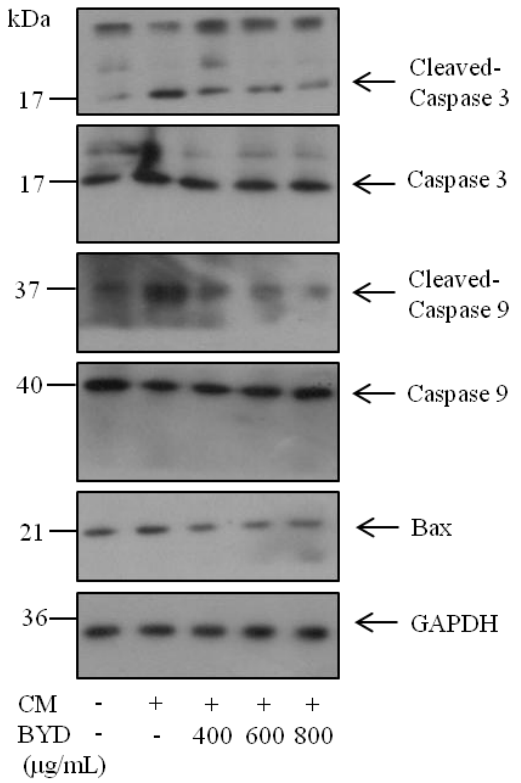

Supplementary Figure 3: Full gel images for Figure 6

Figure 6A

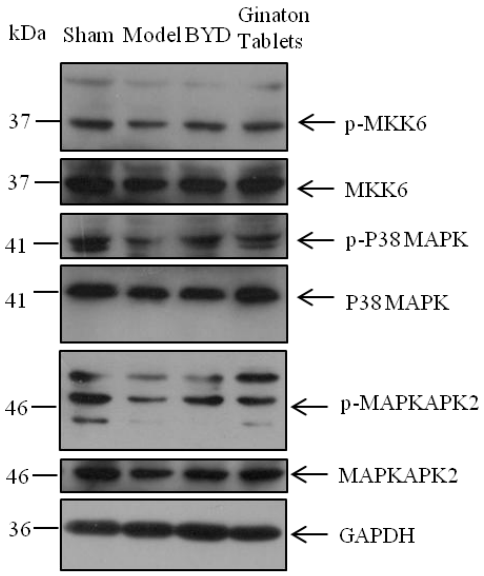

Figure 6C

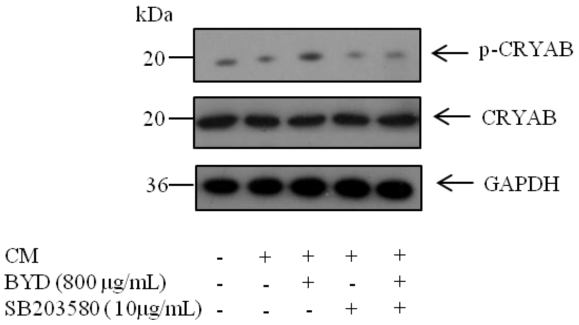

Supplement: Supplementary file 1 [file Presentation_1.PDF]
